# Examining Health-Related Quality of Life in Cancer Survivors: Cross-Sectional Associations with Comorbidities, Navigation Services Use, and Perceived Social Support

**DOI:** 10.3390/cancers17233784

**Published:** 2025-11-26

**Authors:** Daniela López-Vallejo, Cynthia M. Pérez, Lorena González-Sepúlveda, Marievelisse Soto-Salgado

**Affiliations:** 1 Department of Biostatistics and Epidemiology, Graduate School of Public Health, University of Puerto Rico, Medical Sciences Campus, PO Box 365067, San Juan 00936-5067, Puerto Rico; daniela.lopez5@upr.edu (D.L.-V.); cynthia.perez1@upr.edu (C.M.P.); 2 Division of Cancer Control and Population Sciences, University of Puerto Rico, Comprehensive Cancer Center, PO Box 363027, San Juan 00936-3027, Puerto Rico; lorena.gonzalez2@upr.edu

**Keywords:** Hispanics, cancer survivors, health-related quality of life, comorbidities, patient navigation services, social support

## Abstract

**Background/Objectives:** Cancer is the second leading cause of death in Puerto Rico, its impact worsened by an aging population. Many survivors experience long-term effects that reduce health-related quality of life (HRQoL). Chronic comorbidities are common in Puerto Rico, yet cancer survivors remain underrepresented in HRQoL research, raising concern about their impact on survivorship. This study examined whether comorbidity burden was associated with HRQoL and if patient navigation services or perceived social support moderated this association. **Methods:** This cross-sectional analysis included 643 cancer survivors from the START-PR study (November 2023–August 2025). HRQoL was measured using the Functional Assessment of Cancer Therapy—General (FACT-G) and dichotomized at the sample median (≤71 = poor; >71 = non-poor). Comorbidity burden was grouped as 0, 1, or ≥2 conditions. Patient navigation was assessed by self-reported service use in the past year. Perceived social support was measured using an adapted Multidimensional Scale of Perceived Social Support and dichotomized at the sample median (≤40 = low; >40 = high). Logistic regression estimated odds ratios (OR; 95% CI), adjusting for covariates. Interaction terms tested effect modification. **Results:** Participants with one (OR = 1.85; 95% CI: 1.15–2.97) or ≥2 comorbidities (OR = 2.95; 95% CI: 1.88–4.61) had significantly higher odds of poor HRQoL than those without comorbidities. Hypertension, depression, diabetes, arthritis, and asthma were more common among participants with poor HRQoL. Neither patient navigation nor perceived social support significantly moderated the comorbidity burden-HRQoL association. **Conclusions:** Greater comorbidity burden was associated with poor HRQoL. These findings underscore the need for survivorship care models that integrate chronic disease management, including mental health, to improve outcomes in underserved populations.

## Simple Summary

While cancer survivors are living longer, many also face ongoing chronic health conditions that reduce their quality of life. These chronic conditions are common but remain understudied in cancer survivors, particularly in Puerto Rico. This study examined the association between comorbidity burden and health-related quality of life (HRQoL), and whether support services, such as patient navigation or perceived social support, modify this association. Findings showed that cancer survivors with at least one comorbidity were more likely to report poor HRQoL, and that support services did not significantly modify this association. These results highlight the need for survivorship cancer care planning that not only supports cancer recovery but also addresses the management of coexisting chronic illnesses, including mental health conditions. Such research could inform better models of follow-up care that meet the full spectrum of health needs among cancer survivors.

## 1. Introduction

The most recent data from the Puerto Rico Central Cancer Registry indicate that 16,512 new cancer cases were reported in 2022. Moreover, based on data from the 2018–2022 period, an estimated 40% of people in Puerto Rico will be diagnosed with cancer at some point in their lives [1]. Because cancer incidence and mortality rise with age [2], and Puerto Rico is experiencing a rapid demographic shift toward an older population [3], the public health burden of cancer is expected to intensify in the coming decades. While advances in cancer treatment have improved survival, the long-term consequences of cancer and its management take their toll far beyond longevity. Health-related quality of life (HRQoL) has emerged as a critical measure of survivorship [4]; poor HRQoL not only reflects unmet patient needs but is also associated with increased mortality risk [5]. In Puerto Rico, cancer survivors face many persistent psychological, social, and financial stressors that can impact HRQoL; this may hinder recovery and adaptation [6,7]. Despite these challenges, Hispanic/Latino populations, particularly Puerto Ricans living on the island, remain severely underrepresented in HRQoL research [4,5,6,7,8], limiting the evidence base needed to address health inequities.

Comorbid conditions add an additional layer of vulnerability. Between 50% and 80% of cancer patients live with at least one comorbidity, and each additional condition is associated with lower HRQoL [9,10,11,12,13,14,15,16,17]. However, much of the existing research has been conducted in White or Asian cohorts and often centers on a single cancer type, providing limited insight into the lived experiences of Hispanic/Latino survivors. This evidence gap is particularly pressing in Puerto Rico, where comorbidity patterns are shaped by longstanding disparities in chronic disease prevalence [18], and where ongoing fiscal and healthcare system crises further compromise continuity of care and long-term survivorship support. Without population-specific evidence, existing models of survivorship care may fail to capture the full extent of challenges faced by Puerto Rican cancer patients.

Interventions to reduce cancer disparities are urgently needed. Patient navigation has shown promise in narrowing equity gaps by addressing barriers to care, improving treatment adherence, and supporting patients’ psychosocial needs [19,20]. However, evidence regarding its impact on HRQoL, particularly in populations burdened by comorbidities, remains inconclusive [19,20,21]. Similarly, social determinants of health, such as social support, may buffer the negative effects of comorbidities on HRQoL [22]. Supportive networks can mitigate stress, promote healthy behaviors, and strengthen engagement with healthcare systems [22,23], with higher social support linked to better HRQoL [24] and improved adaptation to chronic illness [25]. Nonetheless, the moderating role of perceived social support in cancer survivorship has been scarcely examined among Hispanic/Latino populations.

Given these gaps, this study aimed to assess the association between comorbidity patterns and HRQoL among cancer survivors in Puerto Rico and evaluate whether patient navigation services and perceived social support moderate this association. We found that the presence of comorbidities is associated with poor HRQoL, but neither patient navigation nor perceivedsocial support significantly moderated this association. By focusing on a historically underrepresented and medically vulnerable population, this work seeks to generate urgently needed evidence to inform culturally relevant survivorship strategies, strengthen health system responses, and reduce inequities in cancer outcomes.

## 2. Materials and Methods

### 2.1. Study Design and Sample

We conducted a cross-sectional analysis using data from the START-PR study, the University of Puerto Rico Comprehensive Cancer Center’s first initiative to examine how social determinants of health influence access to the cancer care continuum among cancer patients in Puerto Rico, specifically diagnosis, treatment, and survivorship. Data were collected between November 2023 and August 2025. Eligible participants were ≥21 years old, resided in Puerto Rico, and had received active cancer treatment at any time within the prior 12 months. Of 871 respondents, 228 (26.2%) were excluded due to missing covariate data, yielding a final analytic sample of 643 cancer survivors.

Multiple recruitment strategies were used, including both active methods (e.g., study promotion by trained research assistants in the waiting rooms of three clinics in the San Juan Metropolitan area, outreach by trained research assistants, participation in community events and health fairs, and referrals from family, friends, or healthcare providers) and passive methods (e.g., social media platforms and flyers placed in clinic waiting rooms). Using multiple strategies allowed individuals from across the island to learn about the study and participate if they met the inclusion criteria. Participants completed a confidential 65-question online survey using REDCap, a secure, web-based platform hosted at the University of Puerto Rico Medical Sciences Campus. REDCap supports validated data capture, audit trails, automated export to statistical packages, and integration with external sources [26].

### 2.2. Measures

#### 2.2.1. Health-Related Quality of Life

HRQoL was measured with the Functional Assessment of Cancer Therapy—General (FACT-G) questionnaire, which assesses four domains of well-being: physical, social, emotional, and functional [27]. Total possible scores range from 0 to 108, with higher scores indicating better HRQoL. Because no universally established cut-off value exists for FACT-G scores [28], and the distribution in our sample was skewed, we dichotomized the total FACT-G score at the sample median. Scores ≤ 71 were classified as poor quality of life, and scores > 71 were classified as non-poor, consistent with approaches used in previous studies [29,30]. Internal consistency was excellent (Cronbach’s α = 0.92).

#### 2.2.2. Comorbidity Burden

Comorbidity burden was assessed using a study-specific checklist of commonly self-reported conditions, including diabetes, hypertension, heart disease, lung disease, kidney disease, depression, asthma, lupus, arthritis, chronic obstructive pulmonary disease, anxiety, autoimmune hepatitis, sleep apnea, human immunodeficiency virus, rheumatoid arthritis, hypercholesterolemia, and hypertriglyceridemia [31]. Participants were grouped into three categories, namely zero, one, or two or more comorbidities, balancing clinical interpretability with statistical power.

#### 2.2.3. Patient Navigation Utilization

Patient navigation utilization was assessed with a self-reported yes/no item from the START-PR questionnaire, which asks whether participants had received such services or assistance in the past 12 months. While this single-item measure does not capture all dimensions of patient navigation, it reflects whether survivors accessed assistance during cancer care [32].

#### 2.2.4. Perceived Social Support

Perceived social support was assessed using an adapted version of the Multidimensional Scale of Perceived Social Support (MSPSS), previously adapted and validated by Pérez-Villalobos et al. [33]. This version employs a Spanish translation and a reduced-frequency scale with fewer options, yielding total scores ranging from 12 to 48 [33]. For analysis, scores were dichotomized at the sample median: scores of ≤40 were classified as low perceived social support, and scores of >40 were classified as high perceived social support, given the skewed score distribution. The adapted scale version demonstrated excellent internal consistency (Cronbach’s α = 0.94).

#### 2.2.5. Covariates

Covariates included age at cancer diagnosis, sex, education, cancer stage at diagnosis, physical activity in the past 30 days, residential area, lifetime smoking status, alcohol use in the past 30 days, marital status, time since cancer diagnosis, current cancer treatment status, and history of multiple cancer diagnoses. These variables were selected based on prior research as potential confounders of the relationship between comorbidities and HRQoL [34].

### 2.3. Statistical Analysis

Baseline characteristics were compared by HRQoL status using Student’s *t*-tests or Kruskal–Wallis test for continuous variables and Chi-square tests for categorical variables. Multivariable logistic regression models were employed to estimate odds ratios (ORs) and 95% confidence intervals (CIs) for the association between comorbidity burden and HRQoL, adjusting for all covariates. Interaction terms were introduced to assess whether patient navigation utilization or perceived social support modified the relationship between comorbidity burden and HRQoL.

Sensitivity analyses were conducted to compare participants included in the final analytic sample with those excluded due to missing data, to assess potential bias. Additional analyses assessed whether multivariable model results were consistent when including or excluding patients with multiple cancers. To assess the robustness of study findings, all multivariable models were also re-estimated using HRQoL as a continuous outcome instead of the binary outcome. Model fit was assessed using the Hosmer–Lemeshow test. Variance inflation factors (VIFs) were calculated to evaluate multicollinearity. Analyses were conducted in Stata Software: Release 18.5 (StataCorp LLC, College Station, TX, USA), with a two-sided *p*-value < 0.05 considered statistically significant.

## 3. Results

Participants were primarily women (71.7%), aged 40–64 years at cancer diagnosis (55.4%), with education beyond high school (73.6%), living in urban areas (56.3%), and diagnosed at a localized stage (63.4%) (Table 1). The majority of participants (84.1%) learned about the study through active recruitment methods, most often through a research assistant. Significant differences in sociodemographic, lifestyle, and clinical characteristics by HRQoL status were observed. Poor HRQoL was more frequent among participants with a distant cancer stage, no reported physical activity, multiple cancer diagnoses, and those who did not use patient navigation services. Participants with poor HRQoL also reported lower levels of perceived social support.

**Figure 1:**
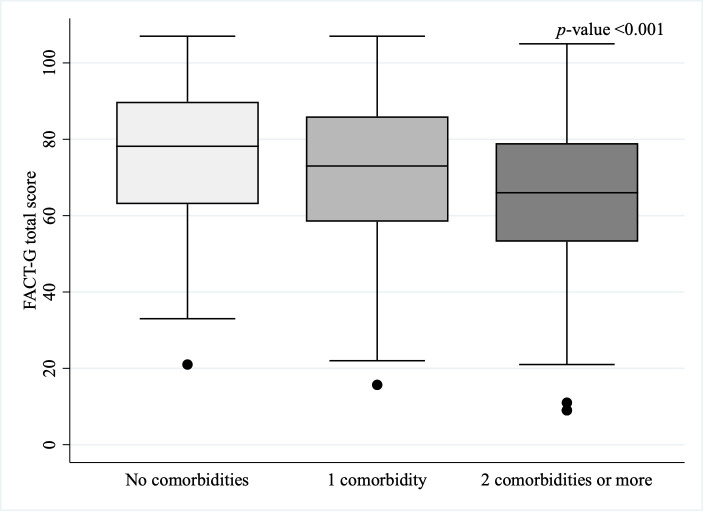
FACT-G total scores by comorbidity burden among cancer survivors receiving care in Puerto Rico, START-PR (*n* = 643).

**Figure 2:**
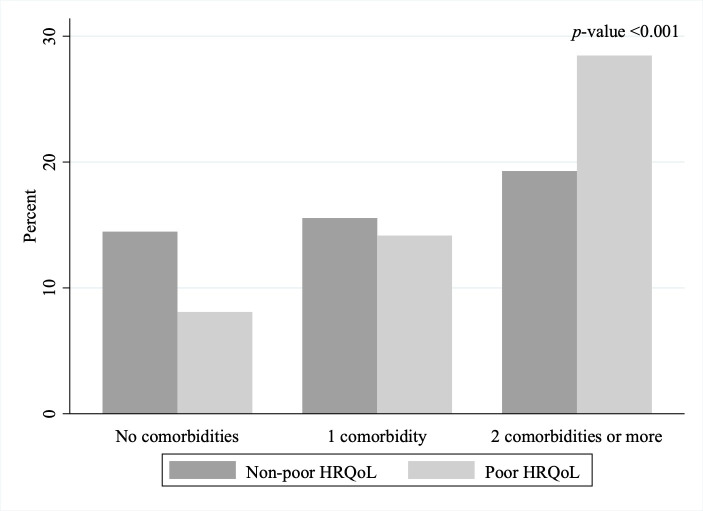
HRQoL by comorbidity burden among cancer survivors receiving care in Puerto Rico, START-PR (*n* = 643).

The most frequently reported comorbidities were hypertension, diabetes, depression, arthritis, and asthma (Table 2), all of which were significantly more prevalent among participants with poor HRQoL. Notably, depression ranked third among those with poor HRQoL but fifth among those with non-poor HRQoL. Median FACT-G scores declined with greater comorbidity burden, with significantly lower scores among those with two or more comorbidities (*p* < 0.001; Figure 1). Similarly, participants reporting ≥ 2 comorbidities were more likely to have poor HRQoL (*p* < 0.001; Figure 2).

In unadjusted logistic regression models, participants with one comorbidity (OR = 1.63, 95% CI: 1.05–2.53) and those with ≥2 comorbidities (OR = 2.64, 95% CI: 1.75–3.97) had significantly higher odds of poor HRQoL compared with those without comorbidities (Table 3). After adjustment for relevant covariates, the association remained significant for one comorbidity (OR = 1.85; 95% CI: 1.15–2.97) and for ≥2 comorbidities (OR = 2.95; 95% CI: 1.88–4.61).

Table 4 presents subgroup analyses of comorbidity burden and poor HRQoL stratified by patient navigation use and perceived social support. Among participants who did not use patient navigation services, both one (OR = 1.92; 95% CI: 1.09–3.38) and ≥2 comorbidities (OR = 3.33; 95% CI: 1.94–5.70) were significantly associated with poor HRQoL. Among those who used patient navigation services, the association was weaker and only significant for those with ≥2 comorbidities (OR = 2.65; 95% CI: 1.05–6.69). A similar pattern was observed by perceived social support: ≥2 comorbidities were significantly associated with poor HRQoL in both low (OR = 3.38, 95% CI: 1.60–7.15) and high support groups (OR = 2.24, 95% CI: 1.18–4.24), with stronger effects in the low support group. Neither patient navigation (*p* = 0.866) nor perceived social support (*p* = 0.535) moderated the comorbidity-HRQoL association.

Comparisons between excluded and included participants revealed differences only in alcohol use during the past 30 days, perceived social support, and poor HRQoL. Sensitivity analyses excluding participants with multiple cancer diagnoses yielded results consistent with the main findings. Additional models adjusting only for covariates significant in the bivariate analyses produced similar results to those adjusting for all covariates identified in the literature. Likewise, linear regression treating HRQoL as a continuous variable showed findings consistent with those from the logistic regression, indicating that a higher comorbidity burden was associated with lower FACT-G scores (mean VIF = 1.41). The Hosmer–Lemeshow test indicated adequate model fit (*p* = 0.327).

## 4. Discussion

This study broadens the literature on the association between comorbidity burden and HRQoL among cancer survivors. Although the association between the two has been determined in other populations, it has not been thoroughly examined in Puerto Rico. Our findings show that greater comorbidity burden is associated with higher odds of poor HRQoL in a population of cancer survivors in Puerto Rico, highlighting the need for integrative survivorship care models that address chronic conditions, particularly in underserved Hispanic/Latino populations.

Consistent with previous studies, participants in our sample exhibited a high comorbidity burden, with 77.4% reporting at least one chronic condition other than cancer. This aligns with prevalence estimates ranging from 70% to 82% among breast and prostate cancer survivors in other studies [9,11,16,35]. The most frequently reported comorbidities were hypertension, diabetes, arthritis, asthma, and depression. Hypertension, diabetes, and arthritis have also been identified as common conditions in prior studies [9,11,36,37]. Unlike prior studies, our results showed a high prevalence of asthma and depression, particularly among those with poor HRQoL, highlighting the need to integrate mental health services into cancer survivorship care planning. Although most prior studies employed linear regression models or correlation analyses, their conclusions are consistent with ours: a higher number of comorbidities is associated with lower HRQoL [9,11,15,16]. These findings emphasize the importance of incorporating comorbidity management into survivorship care in ways that address the specific needs of Puerto Rican cancer survivors.

This study addresses another existing knowledge gap by assessing whether patient navigation use modifies the association between comorbidity burden and HRQoL among cancer survivors in Puerto Rico. Although patient navigation did not significantly moderate this association, the stratum-specific odds ratios were slightly smaller among survivors who used navigation services, suggesting a modest but non-significant trend. Previous studies on patient navigation in other regions have yielded mixed results. For example, a navigation program in the United States significantly improved HRQoL in Latina breast cancer survivors [20], whereas Ramirez et al. [21] found no significant effects among breast and prostate cancer survivors. Our inclusion of multiple cancer types may help explain the differences from previous findings, as differences in supportive care needs across cancer populations have been documented. For example, Latina breast cancer patients have been shown to report higher levels of unmet supportive care needs compared to other groups [38]. In addition, variability in results may be explained by differences in the intensity of patient navigation programs, as some studies have implemented enhanced versions [20,21]. Future studies should evaluate potential moderating effects of patient navigation within specific cancer populations and examine how different program models influence HRQoL.

Although our interaction models did not detect significant moderation, we observed lower odds ratios among participants with higher perceived social support, suggesting a possible buffering effect that warrants further study. Perceived social support nonetheless showed a clear direct association with HRQoL. In bivariate analyses, participants with non-poor HRQoL reported higher perceived social support, consistent with prior research linking greater social support to better HRQoL [24]. Previous studies of Latina breast cancer survivors have shown that percieved social support accounts for roughly 15% of the variance in HRQoL, underscoring its importance [39]. A comparative study of Latina and Caucasian cancer survivors also found racial and ethnic differences in perceived social support, with Caucasian survivors reporting higher percieved social support; psychiatric comorbidities and educational attainment contributed to these disparities [40]. Additional research is needed to clarify how social support may buffer the impact of comorbidity burden on HRQoL among Latino cancer survivors.

This study has several limitations that should be considered. First, the cross-sectional design precludes causal inference. Second, recruitment through clinics, social media, and community events may have favored survivors who are more engaged in healthcare or who have greater digital access, introducing potential selection bias. About one-quarter of participants were excluded due to missing data. Although sensitivity analyses showed consistent results, this exclusion may limit generalizability. Third, patient navigation was measured with a single yes/no item, which may not capture its multidimensional nature. Additionally, although the survey included a description of patient navigation, some participants may not have accurately identified whether they received these services. Fourth, stratified analysis by cancer type produced unstable model estimates with excessively large standard errors due to small sample sizes across several cancer categories, preventing reliable cancer-type-specific modeling. Finally, comorbidity burden was self-reported and categorized broadly, which may introduce misclassification; moreover, the study design does not allow assessment of the temporal sequence between cancer diagnosis and comorbidity onset or the potential reversibility of some reported conditions.

Despite these limitations, our study has notable strengths. We used validated instruments to assess HRQoL (FACT-G) and perceived social support (MSPSS), both of which demonstrated excellent internal reliability. Our models adjusted for a comprehensive set of covariates aligned with prior survivorship research [33]. By examining the combined role of comorbidities, patient navigation, and perceived social support, this study contributes important evidence on how factors beyond cancer itself shape survivorship outcomes, even though we found no evidence that patient navigation or perceived support services moderated these associations. Additionally, this study updates existing knowledge on HRQoL among cancer patients undergoing active treatment in Puerto Rico, yielding results that are consistent with previous findings [30,41].

## 5. Conclusions

Having at least one comorbidity was significantly associated with poor HRQoL among cancer survivors in Puerto Rico, even after adjusting for relevant covariates. Hypertension, diabetes, depression, arthritis, and asthma were more common among participants with poor HRQoL, with hypertension being the most common frequently reported condition in both groups.

These findings underscore the importance of integrating chronic disease management—such as screening for hypertension, depression, and diabetes—into survivorship care as part of the cancer control continuum, to better identify cancer survivors at risk for poor HRQoL in Puerto Rico. In this context, patient navigation services may serve as a critical tool for managing the complex social and clinical needs of cancer survivors with multiple chronic conditions. While the connection between patient navigation and HRQoL in cancer patients with comorbidities is still unclear, these services can help individuals navigate fragmented healthcare systems, facilitate timely referrals, support adherence to both cancer and chronic disease care, and enhance care conditions and overall satisfaction. Future research should explore the individual contributions of common comorbidities to HRQoL and examine the temporal association between comorbidity burden and HRQoL, as well as how patient navigation can be optimized to more effectively address multimorbidity and HRQoL in cancer survivorship care.

## Figures and Tables

**Table 1:** Baseline sociodemographic, lifestyle, and clinical characteristics of cancer survivors receiving care in Puerto Rico, START-PR (*n* = 643).

Variables	Total (*n* = 643)	Not Poor HRQoL (*n* = 317)	Poor HRQoL (*n* = 326)	*p*-Value
Age at diagnosis				0.304
<40 years	63 (9.8%)	32 (50.8%)	31 (49.2%)	
40–64 years	356 (55.4%)	166 (46.6%)	190 (53.4%)	
≥65 years	224 (34.8%)	119 (53.1%)	105 (46.9%)	
Sex at birth				0.514
Female	461 (71.7%)	231 (50.1%)	230 (49.9%)	
Male	182 (28.3%)	86 (47.3%)	96 (52.7%)	
Residence area				0.367
Urban	362 (56.3%)	187 (51.7%)	175 (48.3%)	
Suburban	81 (12.6%)	39 (48.2%)	42 (51.8%)	
Rural	200 (31.1%)	91 (45.5%)	109 (54.5%)	
Education				0.053
High school or less	170 (26.4%)	73 (42.9%)	97 (57.1%)	
More than high school	473 (73.6%)	244 (51.6%)	229 (48.4%)	
Marital status				0.584
Married/lives with partner	347 (54.0%)	171 (49.3%)	176 (50.7%)	
Separated/widowed/divorced	205 (31.9%)	97 (47.3%)	108 (52.7%)	
Single/never married	91 (14.1%)	49 (53.8%)	42 (46.2%)	
Cancer stage				0.006
Localized	408 (63.4%)	220 (53.9%)	188 (46.1%)	
Regional	136 (21.2%)	62 (45.6%)	74 (54.4%)	
Distant	77 (12.0%)	26 (33.8%)	51 (66.2%)	
Unknown	22 (3.4%)	9 (40.9%)	13 (59.1%)	
Comorbidities				<0.001
0	145 (22.6%)	93 (64.1%)	52 (35.9%)	
1	191 (29.7%)	100 (52.4%)	91 (47.6%)	
≥2	307 (47.7%)	124 (40.4%)	183 (59.6%)	
Physical activity (last 30 days)				<0.001
No	429 (66.7%)	178 (41.5%)	251 (58.5%)	
Yes	214 (33.3%)	139 (64.9%)	75 (35.1%)	
Smoking status				0.509
Non-smoker	504 (78.4%)	254 (50.4%)	250 (49.6%)	
Past smoker	110 (17.1%)	51 (46.4%)	59 (53.6%)	
Current smoker	29 (4.5%)	12 (41.4%)	17 (58.6%)	
Alcohol (last 30 days)				0.213
No	496 (77.1%)	236 (47.6%)	260 (52.4%)	
Moderate drinking	56 (8.7%)	33 (58.9%)	23 (41.1%)	
Exceeded moderate drinking	91 (14.2%)	48 (52.7%)	43 (47.3%)	
Current cancer treatment				0.349
No	91 (14.2%)	49 (53.8%)	42 (46.2%)	
Yes	552 (85.8%)	268 (48.5%)	284 (51.5%)	
Time since diagnosis				0.264
≤5 years	584 (90.8%)	292 (50.0%)	292 (50.0%)	
>5 years	59 (9.2%)	25 (42.4%)	34 (57.6%)	
Multiple cancer diagnoses				0.009
No	433 (67.3%)	229 (52.9%)	204 (47.1%)	
Yes	210 (32.7%)	88 (41.9%)	122 (58.1%)	
Perceived social support mean score	38.8 (8.9)	42.5 (6.9)	35.3 (9.3)	<0.001
Patient Navigation				0.006
No	464 (72.2%)	213 (45.9%)	251 (54.1%)	
Yes	179 (27.8%)	104 (58.1%)	75 (41.9%)	

Note: Values are shown as n with row percentages [n (row %)] or as mean (SD).

**Table 2:** Top five comorbid conditions by HRQoL status and their relative rankings among cancer survivors receiving care in Puerto Rico, START-PR (*n* = 643).

Condition	Non-Poor HRQoL		Poor HRQoL		*p* Value
	Ranking	*n* (%) *	Ranking	*n* (%) *	
Hypertension	1	134 (44.2)	1	169 (55.8)	0.013
Diabetes	2	61 (40.1)	2	91 (59.9)	0.009
Depression	5	32 (26.2)	3	90 (73.8)	<0.001
Arthritis	3	56 (41.8)	4	78 (58.2)	0.048
Asthma	4	47 (37.9)	5	77 (62.1)	0.004

* Percentages are calculated within rows, and conditions are ranked according to the frequency of participants reporting each condition. Comorbid conditions are not mutually exclusive.

**Table 3:** Association between comorbidity burden and poor HRQoL among cancer survivors receiving care in Puerto Rico, START-PR (*n* = 643).

Comorbidity Burden	Unadjusted OR (95% CI)	Adjusted OR * (95% CI)
0	1.00	1.00
1	1.63 (1.05–2.53)	1.85 (1.15–2.97)
≥2	2.64 (1.75–3.97)	2.95 (1.88–4.61)

* Model adjusted by age at cancer diagnosis, sex, education, cancer stage, physical activity, residential area, lifetime smoking status, alcohol use, marital status, time since cancer diagnosis, and current cancer treatment status.

**Table 4:** Subgroup analysis for the association between comorbidity burden and poor HRQoL among cancer survivors receiving care in Puerto Rico, START-PR (*n* = 643).

Variable	No Comorbidities	1 Comorbidity Adjusted OR (95% CI) *	≥2 Comorbidities Adjusted OR (95% CI) *	*p*-Value for Interaction
Patient navigation use				
No	1.00	1.92 (1.09–3.38)	3.33 (1.94–5.70)	0.866
Yes	1.00	1.76 (0.64–4.83)	2.65 (1.05–6.69)	
Perceived social support				
Low	1.00	1.86 (0.88–3.97)	3.38 (1.60–7.15)	0.535
High	1.00	1.12 (0.54–2.33)	2.24 (1.18–4.24)	

* Models adjusted by age at cancer diagnosis, sex, education, cancer stage, physical activity, residential area, lifetime smoking status, alcohol use, marital status, time since cancer diagnosis, and current cancer treatment status.

## Data Availability

The data presented in this study are available upon request from the corresponding author.

## Abbreviations

The following abbreviations are used in this manuscript:

HRQoL	Health-related quality of life
FACT-G	Functional Assessment of Cancer Therapy—General
MSPSS	Multidimensional Scale of Perceived Social Support
